# Logarithmic gap costs decrease alignment accuracy

**DOI:** 10.1186/1471-2105-7-527

**Published:** 2006-12-05

**Authors:** Reed A Cartwright

**Affiliations:** 1 Department of Genetics, University of Georgia, Athens, GA 30602-7223, USA; 2 Bioinformatics Research Center, North Carolina State University, Campus Box 7566, Raleigh, NC 27695-7566, USA

## Abstract

**Background:**

Studies on the distribution of indel sizes have consistently found that they obey a power law. This finding has lead several scientists to propose that logarithmic gap costs, *G *(*k*) = *a *+ *c *ln *k*, are more biologically realistic than affine gap costs, *G *(*k*) = *a *+ *bk*, for sequence alignment. Since quick and efficient affine costs are currently the most popular way to globally align sequences, the goal of this paper is to determine whether logarithmic gap costs improve alignment accuracy significantly enough the merit their use over the faster affine gap costs.

**Results:**

A group of simulated sequences pairs were globally aligned using affine, logarithmic, and log-affine gap costs. Alignment accuracy was calculated by comparing resulting alignments to actual alignments of the sequence pairs. Gap costs were then compared based on average alignment accuracy. Log-affine gap costs had the best accuracy, followed closely by affine gap costs, while logarithmic gap costs performed poorly. Subsequently a model was developed to explain the results.

**Conclusion:**

In contrast to initial expectations, logarithmic gap costs produce poor alignments and are actually not implied by the power-law behavior of gap sizes, given typical match and mismatch costs. Furthermore, affine gap costs not only produce accurate alignments but are also good approximations to biologically realistic gap costs. This work provides added confidence for the biological relevance of existing alignment algorithms.

## Background

Sequence alignments are essential to the study of molecular biology and systematics because they purport to reveal regions in sequences that are homologous. Because sequences gain and lose residues as they evolve, alignments are necessary for revealing such gaps in sequence data. Therefore, researchers usually need to align sequences before they can be studied. For example, most algorithms that construct phylogenetic trees from sequences require a sequence alignment (e.g. [1]). Since alignments are an integral part of many research programs, the quality of the inferences we make from alignments depends on the quality of the alignments themselves (e.g. [2]).

There are two main types of alignment algorithms: local and global. Local alignment algorithms like FASTA [3] and BLAST [4] attempt to align only parts of sequences often avoiding gaps, whereas global alignment algorithms like CLUSTAL [5,6] and MCALIGN [7,8] attempt to align entire sequences, explicitly handling gaps. For this study we will focus on the quality of global alignment algorithms for pairs of sequences.

Most global alignment algorithms fall into two categories: finite state automata (FSA) or hidden Markov models (HMM) [9]. FSA came first and relies on finding an alignment that either maximizes a score function or minimizes a cost function based on specific models of scores or costs [10-15]. Often these models are heuristically optimized using a set of "known" alignments. In contrast, pair HMM, a more recent and more powerful approach, relies on establishing a specific statistical model of sequence alignment, often derived from evolutionary principles [8,16-21]. The advantage of pair HMM techniques is that researchers can leverage the full power of probability to the question of alignments, including both frequentist and Bayesian approaches. Pair HMM even allows researchers to sum across all possible alignments to estimate evolutionary parameters and the use of posterior decoding to characterize alignment ambiguity [9]. The most common way to implement both FSA and HMM for pairwise global alignment is through dynamic programming, which allows researchers to both find the single best alignment as well as sum across all possible alignments. Since it is possible to convert between FSA and HMM approaches [9], we are going to focus on an implementation of a minimum cost FSA; however, we will eventually develop a statistical model to explain our results.

An important observation is that alignment accuracy depends on the assumptions used in picking parameters. Costs (the parameters in our approach) that are based on abiological assumptions are likely to produce bad alignments. For example, if the costs of gaps are less than the cost of a match, then the best alignment for a pair of sequences will say that all residues align with gaps, i.e. the sequence pair is unaligned. Only in a limited number of applications will this be a biologically plausible result. A more prudent concern is how to pick the nature of gap costs because using an abiological model of gap costs can render any heuristic optimization of gap costs worthless. Gap costs are typically based on the affine model, where the cost of a gap of length *k *is *G *(*k*) = *a *+ *bk *[10]. This is a popular approach because affine costs are easy to implement, fast, and efficient. Furthermore, since nucleotides are deleted or inserted in groups, it is biologically plausible that gaps should cost more to create than they do to extend, which can be modeled via affine gap costs.

**Figure 1:**
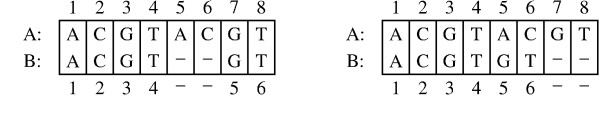
**Example alignment pair**. Numbers identify the residues in the sequences. *k*_1 _columns – A5B-, A6B-, A7B5, and A8B6 – are found in only the left alignment. *K*_2 _columns – A7B-, A8B-, A5B5, and A6B6 – are found in only the right alignment. *K*_3 _columns – A1B1, A2B2, A3B3, and A4B4 – are found in both alignments. Alignment identity is *I *= (*2K*_3_)/(*2K*_3 _+ *K*_1 _+ *K*_2_) = (2 × 4)/(2 × 4 + 4 + 4) = 1/2.

However, some researchers have raised questions about the biological justification for the affine gap model. Studies on the distribution of indel lengths have revealed that the size of an indel is linearly related to its frequency on a log-log scale, and therefore gap-sizes obey a power law [22-26]. Under a Zipfian power-law distribution, the probability that an indel has length *k *is *P*(*k*|*z*) = *k*^-*z*^/*ζ*(*z*), where *z *> 1 and ζ(z)=∑n=1∞n−z
 MathType@MTEF@5@5@+=feaafiart1ev1aaatCvAUfKttLearuWrP9MDH5MBPbIqV92AaeXatLxBI9gBaebbnrfifHhDYfgasaacH8akY=wiFfYdH8Gipec8Eeeu0xXdbba9frFj0=OqFfea0dXdd9vqai=hGuQ8kuc9pgc9s8qqaq=dirpe0xb9q8qiLsFr0=vr0=vr0dc8meaabaqaciaacaGaaeqabaqabeGadaaakeaaiiGacqWF2oGEcqGGOaakcqWG6bGEcqGGPaqkcqGH9aqpdaaeWaqaaiabd6gaUnaaCaaaleqabaGaeyOeI0IaemOEaOhaaaqaaiabd6gaUjabg2da9iabigdaXaqaaiabg6HiLcqdcqGHris5aaaa@3D64@ is Riemann's Zeta function. If 1 <*z *≤ 2, the mean of this distribution is infinite, and if 1 <*z *≤ 3, the variance is infinite. The observation that indel lengths obey a power law suggests that sequences should be aligned using logarithmic gap costs, i.e. *G *(*k*) = *a *+ *c *ln (*k*) [24,25]. However, as mentioned above, the standard method of sequence alignment uses affine gap costs, i.e. *G *(*k*) = *a *+ *bk *because they can be modeled efficiently via Gotoh's algorithm [10]. However, researchers cannot adapt Gotoh's algorithm to logarithmic gap costs, and instead researchers must use the more computationally expensive candidate list method of Waterman [14] as optimized by Miller and Myers [11]. Although affine gap costs are efficient, this study seeks to determine whether this efficiency comes with a cost to accuracy. An alignment is essentially a hypothesis about the evolutionary history of the sequences, specifying formally which residues are homologous to one another. We can define a measurement of alignment accuracy by comparing the hypothesized alignment to the "true" alignment of the sequence pair. An alignment consists of a set of columns which provide per residue homology statements, e.g. residue 100 of sequence A is homologous to residue 90 of sequence B or residue 80 of sequence A is homologous to no residue of sequence B. When comparing two alignment, columns fall into three different categories: 1) columns only appearing in the first alignment, 2) columns only appearing in the second alignment, and 3) columns appearing in both alignments. By counting the number of columns belonging to each category, it is possible to measure how identical two alignments are to one another:

I=2×K32×K3+K1+K2     (1)
 MathType@MTEF@5@5@+=feaafiart1ev1aaatCvAUfKttLearuWrP9MDH5MBPbIqV92AaeXatLxBI9gBaebbnrfifHhDYfgasaacH8akY=wiFfYdH8Gipec8Eeeu0xXdbba9frFj0=OqFfea0dXdd9vqai=hGuQ8kuc9pgc9s8qqaq=dirpe0xb9q8qiLsFr0=vr0=vr0dc8meaabaqaciaacaGaaeqabaqabeGadaaakeaacqWGjbqscqGH9aqpdaWcaaqaaiabikdaYiabgEna0kabdUealnaaBaaaleaacqaIZaWmaeqaaaGcbaGaeGOmaiJaey41aqRaem4saS0aaSbaaSqaaiabiodaZaqabaGccqGHRaWkcqWGlbWsdaWgaaWcbaGaeGymaedabeaakiabgUcaRiabdUealnaaBaaaleaacqaIYaGmaeqaaaaakiaaxMaacaWLjaWaaeWaaeaacqaIXaqmaiaawIcacaGLPaaaaaa@438E@

where *K*_*c *_is the number of columns in category *c*. (See Figure 1 for an example of this measurement.) This alignment identity can be used to measure the accuracy of a hypothesized alignment. It is also possible to describe this formula in hypothesis testing terminology by using true positives, false positives, and false negatives to quantify the accuracy of the hypothesized alignment. In that manner Equation 1 becomes

I=2TP2TP+FP+FN
 MathType@MTEF@5@5@+=feaafiart1ev1aaatCvAUfKttLearuWrP9MDH5MBPbIqV92AaeXatLxBI9gBaebbnrfifHhDYfgasaacH8akY=wiFfYdH8Gipec8Eeeu0xXdbba9frFj0=OqFfea0dXdd9vqai=hGuQ8kuc9pgc9s8qqaq=dirpe0xb9q8qiLsFr0=vr0=vr0dc8meaabaqaciaacaGaaeqabaqabeGadaaakeaacqWGjbqscqGH9aqpdaWcaaqaaiabikdaYiabdsfaujabdcfaqbqaaiabikdaYiabdsfaujabdcfaqjabgUcaRiabdAeagjabdcfaqjabgUcaRiabdAeagjabd6eaobaaaaa@3BB1@

when the first alignment is taken as the hypothesized alignment and the second alignment is taken as the "true" alignment. Unlike the alignment fidelity of Holmes and Durbin [16], alignment identity is symmetric and includes information from gaps.

Not all sequence pairs are equally easy to align, and the accuracy of a hypothesized alignment is expected to decrease as sequence pairs become more distantly related due to substitution saturation and indel accumulation. Therefore, an appropriate measure of expected alignment accuracy for a specific gap cost needs to average across multiple branch lengths and multiple sequence pairs. Branch lengths are often measured in "substitution time", where a unit branch length is equal to 1 substitution, on average, per nucleotide. According to coalescent theory and neutrality, the number of generations separating any pair of sequences in the same diploid population depends on the effective population size, *N*_*e*_, and has approximately an exponential distribution with mean 4*N*_*e *_[27]. If *μ *is the instantaneous rate of substitution per generation, then the substitution time separating any two sequences has an exponential distribution with mean *θ *= 4*N*_*e*_*μ*. As branch lengths get longer and sequences become more distant, data is lost from the sequences, and thus no alignment algorithm may be able to recover the true alignment. This limitation can be corrected on a per-sequence-pair basis by using relative alignment identities: absolute alignment identities divided by the maximum alignment identity found for that sequence pair.

## Results

**Figure 2:**
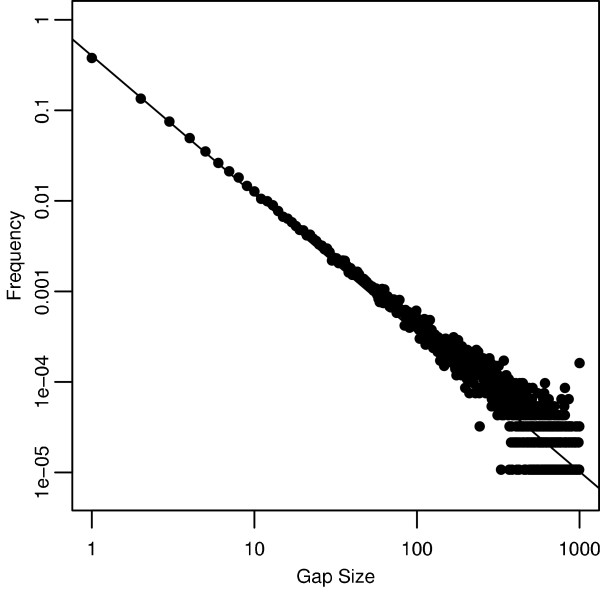
**Gap Sizes Obey a Powerlaw**. Log-Log plot of the distribution of gap sizes measured from the 5000 true alignments. The line is the maximum likelihood fit of a power-law distribution: ln *f *(*k*) = 0.915 – 1.53 ln *k*

For the set of sequence pairs, the minimum branch length for any pair was 1.83 × 10^-05 ^mean substitutions per nucleotide, and the maximum branch length was 1.76. Furthermore, the distribution of observed gap sizes, plotted on a log-log scale, is shown in Figure 2. This distribution clearly obeys a power law. Ignoring the issue of censored data at gap length of 1000, the maximum likelihood estimation of the power-law parameter of this distribution is *z *= 1.53. (The simulation had parameter *z *= 1.5.) Alignments were classified via their parameter values into three different schemes. All parameter sets belonged to the log-affine scheme. The affine and logarithmic schemes were subsets of the log-affine scheme and consisted of the parameter sets where *c *= 0 and *b *= 0, respectively. Analysis of alignment accuracy was divided into two broad and different questions. First, how do the best gap costs for each scheme compare to one another? And second, how do the maximum alignment accuracy for each scheme compare to one another for each sequence pair? The first question investigates what happens if researchers use a single gap cost across many alignments, and the second investigates what happens if researchers optimize gap costs to each alignment.

**Figure 3:**
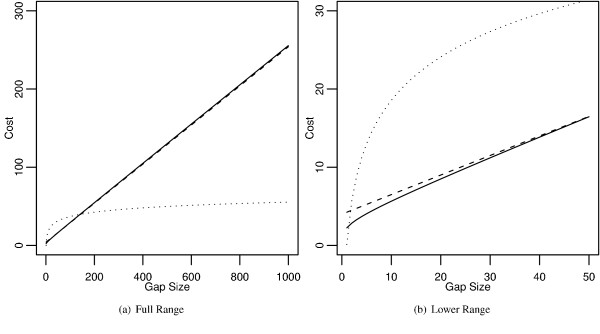
**The curves of the best gap costs**. A) The entire range of the curves and B) a magnification of the beginning of the curves. The best gap costs were decided for each scheme based on highest average alignment identity.  Log-Affine: *G (k) = *2* + k*/4* +* (ln *k)*/2 (solid)* ,* affine* G_A_ (x)* = 4 + *k*/4 (dashed), and logarithmic *G_L_ (k)* = 1/8 + 8 ln k (dotted).

**Figure 4:**
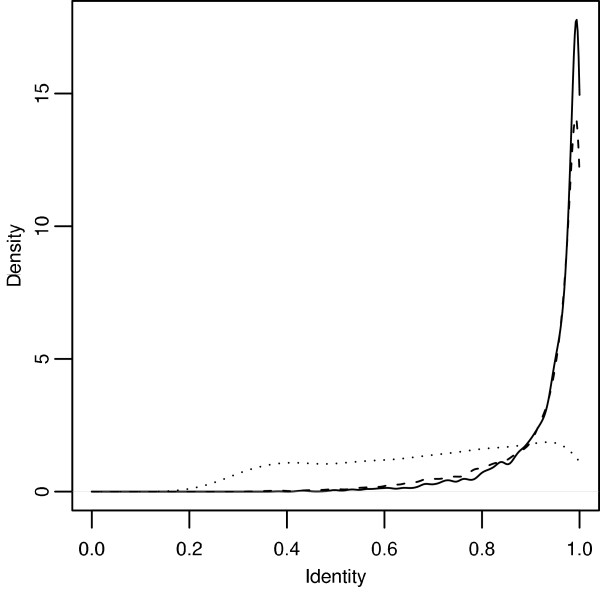
**Accuracy distribution of best gap costs**. Best log-affine (solid), best affine (dashed), and best logarithmic (dotted). Accuracy is measured via alignment identity. See Figure 3 for details on the exact gap costs.

The best gap costs were identified by having the highest average alignment accuracy, i.e. they produced alignments that had the highest average identity to the "true" alignments. The best costs for aligning sequences under the log-affine, affine, and logarithmic schemes were identified respectively as *G *(*k*) = 2 + *k*/4 + (ln *k*)/2 (average identity of 0.941), *G*_*A *_(*k*) = 4 + *k*/4 (average identity of 0.925), and *G*_*L *_(*k*) = 1/8 + 8 ln *k *(average identity of 0.687). Figure 3 shows the graphs of these gap costs, and Figure 4 shows the densities of their identities. Log-affine and affine both peak a little below 100% identity, whereas the logarithmic density is nearly flat for most of the parameter space before barely peaking below 100% identity. Tables 1 and 2 present some statistical properties of these gap penalties. The best log-affine cost produced alignments that were only slightly better than the ones produced by the best affine cost. Both log-affine and affine costs produced alignments that were considerably better than the ones produced by the best logarithmic cost. In fact, the best log-affine gap cost produced the best alignments for over half the sequence pairs.

**Table 1:** Absolute accuracy properties of the best gap costs

	Absolute Identities		
	Log- Affine	Affine	Logarithmic
Minimum	0.383	0.324	0.183
1st Quartile	0.926	0.904	0.512
Mean	0.941	0.925	0.687
Median	0.976	0.970	0.717
3rd Quartile	0.994	0.992	0.874
Maximum	1.0	1.0	1.0

Accuracy is measured via alignment identity. Log-Affine: *G (k) = *2* + k*/4* +* (ln *k)*/2 (solid)* ,* affine* G_A_ (x)* = 4 + *k*/4 (dashed), and logarithmic *G_L_ (k)* = 1/8 + 8 ln k (dotted).

**Table 2:** Relative accuracy properties of the best gap costs

	Relative Identities		
	Log-Affine	Affine	Logarithmic
Minimum	0.710	0.501	0.193
1st Quartile	0.993	0.971	0.549
Mean	0.992	0.973	0.717
Median	1.0	0.993	0.745
3rd Quartile	1.0	1.0	0.892
Maximum	1.0	1.0	1.0

Relative accuracy was calculated as the alignment identity produced by a gap cost for each sequence pair divided by the largest alignment identity produced by any gap cost for the same sequence pair. See notes of Table 1.

**Figure 5:**
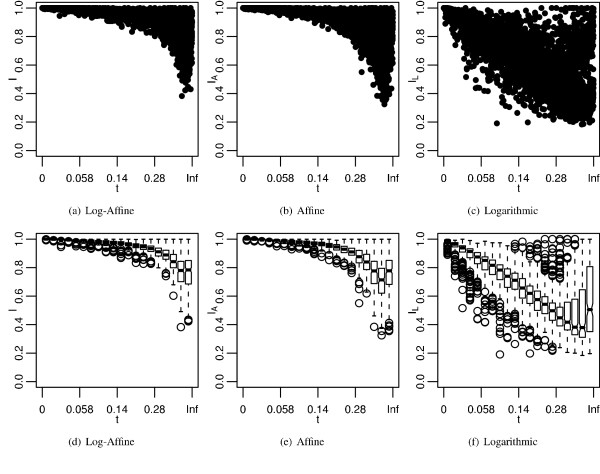
**Accuracies of best costs plotted by divergence**. *I*, *I*_*A*_, and *I*_*L *_are the alignment identities produced by the best log-affine, affine, and logarithmic gap penalties, respectively. See Figure 3 for more information. a-c) Alignment identities plotted by the branch length of the alignments. Divergence time is plotted on a uniform scale, *u *= 1 - exp (-*t*/t¯
 MathType@MTEF@5@5@+=feaafiart1ev1aaatCvAUfKttLearuWrP9MDH5MBPbIqV92AaeXatLxBI9gBaebbnrfifHhDYfgasaacH8akY=wiFfYdH8Gipec8Eeeu0xXdbba9frFj0=OqFfea0dXdd9vqai=hGuQ8kuc9pgc9s8qqaq=dirpe0xb9q8qiLsFr0=vr0=vr0dc8meaabaqaciaacaGaaeqabaqabeGadaaakeaacuWG0baDgaqeaaaa@2E35@). d-f) Box-whisker plots of identities grouped into 20 bins of 250 values. Solid bars are medians. Notches are significant range of medians. Bars are the mid-range. Whiskers are the range. Circles are outliers.

Figure 5 looks at the distribution of identities produced by each best cost. Figure 5a–c plots the identities with respect to their branch lengths, transformed to a uniform scale. Figure 5d–f are box-whisker plots of identities grouped into 20 classes based on branch length. The best logarithmic gap cost produces alignments with much lower identities than the best log-affine and affine costs. As expected, identities decrease as branch lengths increase; however, unexpectedly, the largest branch lengths show increasing alignment identity.

**Figure 6:**
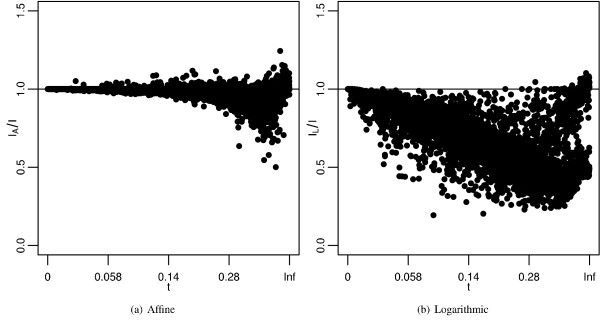
**Accuracies of best costs compared per sequence**. Ratio of identities produced by a) best affine gap cost and b) best logarithmic gap cost to the identities produced by the log-affine gap cost plotted for each sequence pair by divergence time. See Figure 5 for more information.

To compare the best gap costs on a per sequence pair basis, Figure 6 shows the ratio of affine and logarithmic alignment identities to log-affine alignment identities, plotted via branch length for each sequence pair. The identities produced by the best log-affine gap cost tend to be higher than or equal to the identities produced by the best affine and best logarithmic gap costs. However, there are some sequences for which the best log-affine gap cost produces an alignment that is worse than the alignment produced by the best affine or best logarithmic cost. Nevertheless, the best affine cost compares rather well to the best log-affine cost, especially at lower branch lengths. However, the best logarithmic cost does a poor job compared to the best log-affine cost and the best affine cost. Clearly alignments derived from logarithmic costs are of poor quality, and highly sensitive to the divergence between sequences.

**Figure 7:**
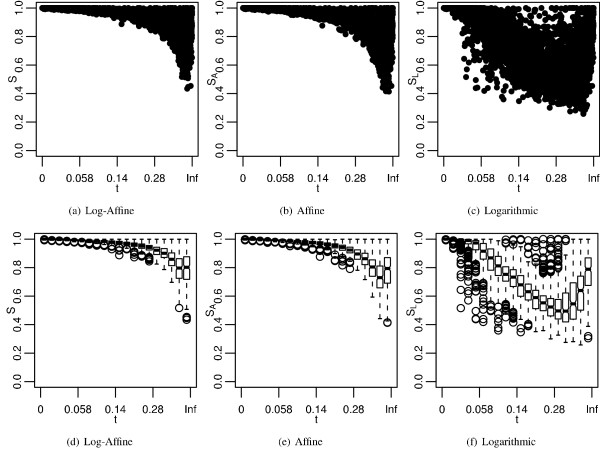
**Maximum accuracies plotted by divergence**. *S*, *S*_*A*_, and *S*_*L *_are the maximum alignment identity produced for each sequence pair by log-affine, affine, and logarithmic gap costs respectively. The subfigures are the same as in Figure 5.

**Figure 8:**
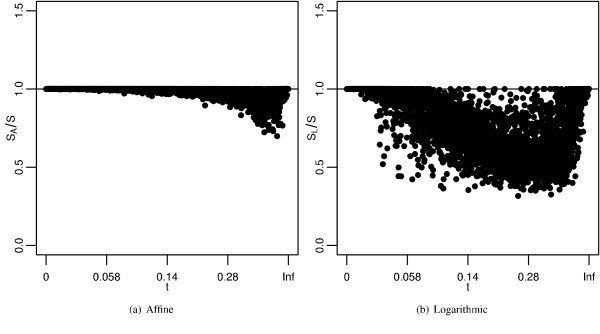
**Maximum accuracies compared per sequence**. Ratio of maximum identities produced by a) affine gap costs and b) logarithmic gap costs to the maximum identities produced by log-affine gap costs plotted for each sequence pair by divergence time. See Figures 5-7 for more information.

Instead of trying to find gap costs that have the highest average accuracy, we can find the gap costs that have the highest accuracy for each sequence pair. Therefore, an alternative approach to comparing schemes is to look at the maximum identity produced by each scheme for each sequence pair. Similar to Figure 5, Figure 7 shows the maximum identities of each scheme plotted by transformed branch length, and box-whisker plots of the data. As we saw in the best cost analysis, the maximum affine identities are similar to maximum log-affine identities, and both are distinct from the maximum logarithmic identities. Identities decrease with increasing branch lengths, only to increase with the largest branch lengths. Furthermore, logarithmic densities are once again very sensitive to increasing branch lengths. Similar to Figure 6, Figure 8 shows the ratio of maximum identities of affine and logarithmic to the log-affine schemes. Once again, the affine scheme has identities similar to the log-affine scheme and the logarithmic scheme does not.

## Discussion

The first issue that we will consider is whether the parameter space was properly sampled. For log-affine and affine schemes, the best values were found inside the sampled parameter space, representing local maxima and perhaps global maxima. However, for logarithmic gap penalties, the best penalty was found on the edge of the parameter space. Subsequent expansion of the parameter space confirmed that *G*_*L *_(*k*) = 1/8 + 8 ln *k *represents a local maximum for logarithmic gap costs.

In the simulations, branch lengths were randomly drawn based on *θ *= 4*N*_*e*_*μ *= 0.2. If the per-nucleotide mutation rate is *μ *= 10^-9^, then the effective population size would be 50 million. This is high for most populations, but it does produce many branch lengths that can represent species-species divergence times. When calculating the best gap costs, it is possible to use importance sampling to weight the identities in a way that reflects another distribution of branch lengths. Similar results (not shown) were obtained when weighting to produce a *θ *= 0.002 distribution.

An interesting feature of the data is that alignment identity improves at the longest branch lengths. According to Figure 5, this occurs after the ninety-fifth percentile, which roughly includes all identities associated with branch lengths greater than 0.6, which is three times the mean branch length. More than likely, branch lengths much larger than 0.6 are responsible for this observation, but Figure 5 does not have the resolution to detect a more precise threshold. The observation that alignment identity improves at the longest branch lengths can be attributed to the fact that sequences at long branch lengths, although related, are saturated with indels and thus have very few nucleotides homologous to one another anymore. Therefore, parameters that tend to produce hypothesized alignments dominated by gaps, which cause low identities elsewhere, show high identity to the true alignment at long branch lengths.

Clearly from the results, logarithmic gap costs are a poor choice for aligning sequences even though biological results would seem to suggest them. Logarithmic gap costs perform poorly because they increase slowly (Figure 3). This causes logarithmic costs to "cheat" during pairwise alignments because two huge gaps, covering the entirety of the sequences may be less costly than three or more moderate gaps. In fact, many logarithmic costs have bimodal distributions; they either work or cheat. However, this may not be a problem because it is easy to tell when logarithmic costs cheat, which can be reflected by posterior decoding [9,18]. Log-affine gap costs are noticeably better than simple affine gap costs, even though the difference may not be enough to justify wide spread usage given the slower speed of the candidate list method. According to the above results, affine gap costs only diverge from log-affine gap penalties at large branch lengths.

It is definitely surprising that logarithmic gap costs do so poorly compared to affine and log-affine gap costs, given that initially there seems to be little biological justification for having a linear component in the gap cost. However, as we show in Appendices A and B, converting a maximum likelihood search into a minimum cost search through shifting and scaling introduces a linear component into the gap cost which can dominate the logarithmic component. In other words, the power law does not imply that gap costs should be logarithmic, instead it implies that gap costs should be log-affine.

In Appendix A we use techniques from the field of statistical alignment to develop a probability model for our alignments. The model is similar to the model of Knudsen and Miyamoto [17]. However, it differs in part by not explicitly treating overlapping indels and using a more realistic Zipf power-law distribution for indel lengths. In contrast to the recent practice of employing a mixed-geometric model [19,28], a power-law model is simpler – one parameter versus three – and has a fatter tail. Also as discussed above, it is more relevant to the observed distribution of indels [22-26]. Our probability model is used to develop a maximum likelihood search for the best alignment and then convert that maximum likelihood search into a minimum cost FSA. Maximum likelihood may not be as powerful as posterior decoding [9,18], but it is easy to convert into a minimum cost FSA.

From Appendix A, the log-likelihood of a pairwise, global alignment given observed sequences *A *and *B *and parameters *λ*, *θ*, and *z *is

ln⁡L(Aln+A,B)=Mln⁡(1+3e−4θ/3)+Rln⁡(1−e−4θ/3)+∑g=1N[ln⁡(eλθ−1)−ln⁡ζ(z)−zln⁡kg]     (2)
 MathType@MTEF@5@5@+=feaafiart1ev1aaatCvAUfKttLearuWrP9MDH5MBPbIqV92AaeXatLxBI9gBaebbnrfifHhDYfgasaacH8akY=wiFfYdH8Gipec8Eeeu0xXdbba9frFj0=OqFfea0dXdd9vqai=hGuQ8kuc9pgc9s8qqaq=dirpe0xb9q8qiLsFr0=vr0=vr0dc8meaabaqaciaacaGaaeqabaqabeGadaaakeaacyGGSbaBcqGGUbGBcqWGmbatcqGGOaakcqWGbbqqieGacqWFSbaBcqWFUbGBcqGHRaWkcqWGbbqqcqGGSaalcqWGcbGqcqGGPaqkcqGH9aqpcqWGnbqtcyGGSbaBcqGGUbGBdaqadaqaaiabigdaXiabgUcaRiabiodaZiabdwgaLnaaCaaaleqabaGaeyOeI0IaeGinaqdcciGae4hUdeNaei4la8IaeG4mamdaaaGccaGLOaGaayzkaaGaey4kaSIaemOuaiLagiiBaWMaeiOBa42aaeWaaeaacqaIXaqmcqGHsislcqWGLbqzdaahaaWcbeqaaiabgkHiTiabisda0iab+H7aXjabc+caViabiodaZaaaaOGaayjkaiaawMcaaiabgUcaRmaaqahabaWaamWaaeaacyGGSbaBcqGGUbGBcqGGOaakcqWGLbqzdaahaaWcbeqaaiab+T7aSjab+H7aXbaakiabgkHiTiabigdaXiabcMcaPiabgkHiTiGbcYgaSjabc6gaUjab+z7a6jabcIcaOiabdQha6jabcMcaPiabgkHiTiabdQha6jGbcYgaSjabc6gaUjabdUgaRnaaBaaaleaacqWGNbWzaeqaaaGccaGLBbGaayzxaaaaleaacqWGNbWzcqGH9aqpcqaIXaqmaeaacqWGobGta0GaeyyeIuoakiaaxMaacaWLjaWaaeWaaeaacqaIYaGmaiaawIcacaGLPaaaaaa@82A9@

where *λ *is the mean number of indels per substitution, *θ *is the average branch length between sequences, and *z *is the power-law parameter. The alignment is summarized by the number of matches (*M*), number of mismatches (*R*), and the length of each gap (*k*_1 _. . . *k*_*N*_). Furthermore, in Appendix B we convert this log-likelihood into minimum cost search, producing the following gap cost derived from Equation 2:

G(k)=ln⁡ζ(z)−ln⁡(eλθ−1)+ln⁡(1+3e−4θ/3)k/2+zln⁡kln⁡(1+3e−4θ/3)−ln⁡(1−e−4θ/3)     (3)
 MathType@MTEF@5@5@+=feaafiart1ev1aaatCvAUfKttLearuWrP9MDH5MBPbIqV92AaeXatLxBI9gBaebbnrfifHhDYfgasaacH8akY=wiFfYdH8Gipec8Eeeu0xXdbba9frFj0=OqFfea0dXdd9vqai=hGuQ8kuc9pgc9s8qqaq=dirpe0xb9q8qiLsFr0=vr0=vr0dc8meaabaqaciaacaGaaeqabaqabeGadaaakeaacqWGhbWrcqGGOaakcqWGRbWAcqGGPaqkcqGH9aqpdaWcaaqaaiGbcYgaSjabc6gaUHGaciab=z7a6jabcIcaOiabdQha6jabcMcaPiabgkHiTiGbcYgaSjabc6gaUjabcIcaOiabdwgaLnaaCaaaleqabaGae83UdWMae8hUdehaaOGaeyOeI0IaeGymaeJaeiykaKIaey4kaSIagiiBaWMaeiOBa4MaeiikaGIaeGymaeJaey4kaSIaeG4mamJaemyzau2aaWbaaSqabeaacqGHsislcqaI0aancqWF4oqCcqGGVaWlcqaIZaWmaaGccqGGPaqkcqWGRbWAcqGGVaWlcqaIYaGmcqGHRaWkcqWG6bGEcyGGSbaBcqGGUbGBcqWGRbWAaeaacyGGSbaBcqGGUbGBcqGGOaakcqaIXaqmcqGHRaWkcqaIZaWmcqWGLbqzdaahaaWcbeqaaiabgkHiTiabisda0iab=H7aXjabc+caViabiodaZaaakiabcMcaPiabgkHiTiGbcYgaSjabc6gaUjabcIcaOiabigdaXiabgkHiTiabdwgaLnaaCaaaleqabaGaeyOeI0IaeGinaqJae8hUdeNaei4la8IaeG4mamdaaOGaeiykaKcaaiaaxMaacaWLjaWaaeWaaeaacqaIZaWmaiaawIcacaGLPaaaaaa@7ED3@

Since in the simulations *θ *= 0.2, *λ *= 0.15, and *z *= 1.5, Equation 2 reduces to

ln⁡L(Aln|A,B)=1.19M−1.45R−∑g=1G[4.45+1.5ln⁡kg]     (4)
 MathType@MTEF@5@5@+=feaafiart1ev1aaatCvAUfKttLearuWrP9MDH5MBPbIqV92AaeXatLxBI9gBaebbnrfifHhDYfgasaacH8akY=wiFfYdH8Gipec8Eeeu0xXdbba9frFj0=OqFfea0dXdd9vqai=hGuQ8kuc9pgc9s8qqaq=dirpe0xb9q8qiLsFr0=vr0=vr0dc8meaabaqaciaacaGaaeqabaqabeGadaaakeaacyGGSbaBcqGGUbGBcqWGmbatcqGGOaakcqWGbbqqieGacqWFSbaBcqWFUbGBcqGG8baFcqWGbbqqcqGGSaalcqWGcbGqcqGGPaqkcqGH9aqpcqaIXaqmcqGGUaGlcqaIXaqmcqaI5aqocqWGnbqtcqGHsislcqaIXaqmcqGGUaGlcqaI0aancqaI1aqncqWGsbGucqGHsisldaaeWbqaaiabcUfaBjabisda0iabc6caUiabisda0iabiwda1iabgUcaRiabigdaXiabc6caUiabiwda1iGbcYgaSjabc6gaUjabdUgaRnaaBaaaleaacqWGNbWzaeqaaOGaeiyxa0faleaacqWGNbWzcqGH9aqpcqaIXaqmaeaacqWGhbWra0GaeyyeIuoakiaaxMaacaWLjaWaaeWaaeaacqaI0aanaiaawIcacaGLPaaaaaa@6162@

and Equation 3 reduces to

*G *(*k*) = 1.69 + 0.23*k *+ 0.56 ln *k *    (5)

This gap cost is very close to the top gap cost found in the simulations: *G *(*k*) = 2 + 0.25*k *+ 0.51n *k*.

Furthermore, based on unweighted least squares, the following affine cost bests fits Equation 5: *G *(*k*) = 4.17 + 0.23*k *(unweighted mean squared error of 0.0722). This cost is very close to the best affine cost found in the simulations, w′k
 MathType@MTEF@5@5@+=feaafiart1ev1aaatCvAUfKttLearuWrP9MDH5MBPbIqV92AaeXatLxBI9gBaebbnrfifHhDYfgasaacH8akY=wiFfYdH8Gipec8Eeeu0xXdbba9frFj0=OqFfea0dXdd9vqai=hGuQ8kuc9pgc9s8qqaq=dirpe0xb9q8qiLsFr0=vr0=vr0dc8meaabaqaciaacaGaaeqabaqabeGadaaakeaacuWG3bWDgaqbamaaBaaaleaacqWGRbWAaeqaaaaa@2FBA@ = 4 + 0.25*k*.

Furthermore, because the linear component of Equation 5 dominates the logarithmic component, logarithmic gap costs will clearly provide worse fits than affine gap costs. Therefore, one can surmise that the linear component to the gap cost function derives from the conversion of a maximum likelihood search into a minimum cost search via shifting and scaling to fit specific substitution costs. Furthermore, this linear component dominates the gap cost allowing the log component to be removed and the gap opening cost re-waited. These results also open the possibility that the gap extension cost can be moved into the substitution matrix and eliminated from the gap cost entirely, potentially speeding up alignment algorithms.

The linear component of the affine approximation is derived solely from the shifting and scaling introduced by fixing the substitution costs. Because the extension cost is not influenced by the distribution of gap lengths, the Zipf power-law distribution of gap sizes appears to be approximated by a discrete uniform distribution. Although this result is rather unexpected, it makes sense in two ways. First, Zipf distributions have fat tails, and sections of the tail can be well approximated by a uniform distribution. And second, the numbers of matches, mismatches, and gapped positions are not independent of one another (Appendix B); therefore, matches and mismatches carry information about gap lengths. The uniform approximation for a Zipf distribution may prove to be more useful than geometric [17,21] or mixed-geometric models [19,28].

## Conclusion

From these results I propose that, if a researcher knows or is willing to assume *θ*, *λ*, and *z *for a group of sequences that he wants to align using a match cost of 0 and a mismatch cost of 1, he can estimate a log-affine gap cost via Equation 3. However, if he wanted to use other costs for matches and mismatches, he can re-derive them using the methodology shown here. Furthermore, an affine gap cost can be estimated by fitting *G *(*k*) = *a *+ *bk *to Equation 3 via the method of least squares. However, researchers will find more utility if the procedure outlined in this paper was extended to the models of sequence evolution beyond Jukes-Cantor. In subsequent research, I hope to apply this procedure to more complex models as well as to unrooted trees.

This research has demonstrated that logarithmic gap costs, although suggested by biological data on the surface, are not a good solution for aligning pairs of sequences through a finite state automata. In fact, despite previous suggestions, e.g. [25], the power law does not imply that gap costs should be logarithmic, instead it implies that gap costs should be log-affine. Furthermore, the results find that affine gap costs can serve as a good approximation to log-affine gap costs to account for the shifting and scaling often introduced by match and mismatch scores. Because affine gap costs are quick, efficient, and currently nearly ubiquitous, this research strengthens the rational for existing practices in molecular biology.

## Methods

Five thousand sequence pairs were generated on unrooted trees using the sequence simulation program, Dawg [29]. Dawg is a sequence simulation program that combines the general time reversible substitution model with a continuous time indel formation model. It is probably the only sequence simulation program capable of natively using the power-law model for indel lengths. Each simulation performed by Dawg started with a random sequence of 1000 nucleotides. For each ancestral sequence, a single descendant sequence was evolved by Dawg based on the branch length separating the ancestor from the descendant. The branch lengths were drawn from an exponential distribution with a mean of *θ *= 0.2. Because sequences were to be aligned using equal costs for each mismatch type, the sequences were evolved under the Jukes-Cantor substitution model [30]. Indels were created at a rate of 15 per 100 substitutions [29], and their lengths were distributed via a truncated power-law with parameter of 1.5 [26] and a cut-off of 1000 nucleotides. The observed distribution of gaps was checked to see if it obeyed a power-law, and the power-law parameter was estimated using maximum likelihood [31]. Dawg recorded the actual alignment of each sequence pair making it possible to measure the accuracy of alignments generated through dynamic programming.

Pairwise, global alignments were done with Ngila [32], an implementation of the candidate-list dynamic programming algorithm of Miller and Myers [11] for logarithmic and affine gap costs. Because gap costs are usually optimized for specific substitution costs [9], the cost of a match was chosen to be 0 and the cost of a mismatch to be 1. Each sequence pair was aligned using 512 different parameter sets, which specified the coefficients of the gap cost function, *G *(*k*) = *a *+ *bk *+ *c *ln *k*. Each coefficient was one of eight values: 0, 1/8, 1/4, 1/2, 1, 2, 4, or 8. The alignment identity (Equation 1) of each of these 2.56 million hypothesized alignments was calculated with respect to the appropriate true alignment produced by Dawg. Expansion of the parameter space to verify the local maximum for logarithmic gap costs used *a *= 16.

The statistical software, R [33], was used to analyze the alignment identities and produce most figures. Fitting affine gap costs to the optimal gap costs was done via the method of least squares for gap sizes 1 to 1000. The squared error was minimized separately using the optimization procedures in PopTools 2.7.1 [34] and Mathematica 5.1 [35].

## Appendices

### A. Alignment log-likelihood

In this appendix we will develop a statistical model for alignment similar to [17] but simpler. To find the most likely alignment we need a measurement of the likelihood of an alignment given the observed pair of sequences, *A *and *B*, and predetermined evolutionary parameters. This likelihood is proportional to the density of the alignment given the sequence pair [36] (p9):

L(Aln|A,B)∝f(Aln|A,B)=f(Aln,A,B)f(A,B)∝f(Aln,A,B)     (6)
 MathType@MTEF@5@5@+=feaafiart1ev1aaatCvAUfKttLearuWrP9MDH5MBPbIqV92AaeXatLxBI9gBaebbnrfifHhDYfgasaacH8akY=wiFfYdH8Gipec8Eeeu0xXdbba9frFj0=OqFfea0dXdd9vqai=hGuQ8kuc9pgc9s8qqaq=dirpe0xb9q8qiLsFr0=vr0=vr0dc8meaabaqaciaacaGaaeqabaqabeGadaaakeaacqWGmbatcqGGOaakcqWGbbqqieGacqWFSbaBcqWFUbGBcqGG8baFcqWGbbqqcqGGSaalcqWGcbGqcqGGPaqkcqGHDisTcqWGMbGzcqGGOaakcqWGbbqqcqWFSbaBcqWFUbGBcqGG8baFcqWGbbqqcqGGSaalcqWGcbGqcqGGPaqkcqGH9aqpdaWcaaqaaiabdAgaMjabcIcaOiabdgeabjab=XgaSjab=5gaUjabcYcaSiabdgeabjabcYcaSiabdkeacjabcMcaPaqaaiabdAgaMjabcIcaOiabdgeabjabcYcaSiabdkeacjabcMcaPaaacqGHDisTcqWGMbGzcqGGOaakcqWGbbqqcqWFSbaBcqWFUbGBcqGGSaalcqWGbbqqcqGGSaalcqWGcbGqcqGGPaqkcaWLjaGaaCzcamaabmaabaGaeGOnaydacaGLOaGaayzkaaaaaa@663F@

To calculate Equation 6 completely for two sequences related by a common ancestor, one would have to consider all sequences that could be the most recent common ancestor of A and B and all possible branch lengths between this ancestor and *A *and *B*. However, our simulations assumed that the tree relating *A *and *B *was unrooted, and thus *A *was considered to be descended from *B*, eliminating the need to consider the set of all possible progenitors for both sequences. We calculate Equation 6 based on the evolutionary distance or branch length *t *between sequences *A *and *B*:

f(Aln,A,B)=∫tf(Aln,A,B,t)dt     (7)
 MathType@MTEF@5@5@+=feaafiart1ev1aaatCvAUfKttLearuWrP9MDH5MBPbIqV92AaeXatLxBI9gBaebbnrfifHhDYfgasaacH8akY=wiFfYdH8Gipec8Eeeu0xXdbba9frFj0=OqFfea0dXdd9vqai=hGuQ8kuc9pgc9s8qqaq=dirpe0xb9q8qiLsFr0=vr0=vr0dc8meaabaqaciaacaGaaeqabaqabeGadaaakeaacqWGMbGzcqGGOaakcqWGbbqqieGacqWFSbaBcqWFUbGBcqGGSaalcqWGbbqqcqGGSaalcqWGcbGqcqGGPaqkcqGH9aqpdaWdraqaaiabdAgaMjabcIcaOiabdgeabjab=XgaSjab=5gaUjabcYcaSiabdgeabjabcYcaSiabdkeacjabcYcaSiabdsha0jabcMcaPaWcbaGaemiDaqhabeqdcqGHRiI8aOGaemizaqMaemiDaqNaaCzcaiaaxMaadaqadaqaaiabiEda3aGaayjkaiaawMcaaaaa@4F6E@

It is possible to derive Equation 7 from an evolutionary process. Specifically the probability that *B *gave rise to *A *over evolutionary distance *t *with indels to produce alignment *Aln*.

*f *(*Aln*, *A*, *B*, *t*) = *f *(*B *→ *A*, *t*, *Aln*) = *f *(*A*|*Aln*, *B*, *t*) *f *(*Aln*|*B*, *t*) *f *(*t*) *f *(*B*)     (8)

where *f*(*t*) = exp (-t/*θ*)/*θ *is the density of branch lengths between *A *and *B *and f(B)=4−Lb
 MathType@MTEF@5@5@+=feaafiart1ev1aaatCvAUfKttLearuWrP9MDH5MBPbIqV92AaeXatLxBI9gBaebbnrfifHhDYfgasaacH8akY=wiFfYdH8Gipec8Eeeu0xXdbba9frFj0=OqFfea0dXdd9vqai=hGuQ8kuc9pgc9s8qqaq=dirpe0xb9q8qiLsFr0=vr0=vr0dc8meaabaqaciaacaGaaeqabaqabeGadaaakeaacqWGMbGzcqGGOaakcqWGcbGqcqGGPaqkcqGH9aqpcqaI0aandaahaaWcbeqaaiabgkHiTiabdYeamnaaBaaameaacqWGIbGyaeqaaaaaaaa@3671@ is the probability for ancestral sequence *B *of length *L*_*b*_. If *L*_*a*_, *M*, and *R *are respectively the length of sequence *A*, the number of matches in the alignment, and the number of replacements, then under the Jukes-Cantor model,

f(A|Aln,t,B)=4−La(1+3e−4t/3)M(1−e−4t/3)R
 MathType@MTEF@5@5@+=feaafiart1ev1aaatCvAUfKttLearuWrP9MDH5MBPbIqV92AaeXatLxBI9gBaebbnrfifHhDYfgasaacH8akY=wiFfYdH8Gipec8Eeeu0xXdbba9frFj0=OqFfea0dXdd9vqai=hGuQ8kuc9pgc9s8qqaq=dirpe0xb9q8qiLsFr0=vr0=vr0dc8meaabaqaciaacaGaaeqabaqabeGadaaakeaacqWGMbGzcqGGOaakcqWGbbqqcqGG8baFcqWGbbqqieGacqWFSbaBcqWFUbGBcqGGSaalcqWG0baDcqGGSaalcqWGcbGqcqGGPaqkcqGH9aqpcqaI0aandaahaaWcbeqaaiabgkHiTiabdYeamnaaBaaameaacqWGHbqyaeqaaaaakmaabmaabaGaeGymaeJaey4kaSIaeG4mamJaemyzau2aaWbaaSqabeaacqGHsislcqaI0aancqWG0baDcqGGVaWlcqaIZaWmaaaakiaawIcacaGLPaaadaahaaWcbeqaaiabd2eanbaakmaabmaabaGaeGymaeJaeyOeI0Iaemyzau2aaWbaaSqabeaacqGHsislcqaI0aancqWG0baDcqGGVaWlcqaIZaWmaaaakiaawIcacaGLPaaadaahaaWcbeqaaiabdkfasbaaaaa@57E2@

The probability that an indel occurs at any position is 1 - *e*^-*λt*^, and, if we ignore the issue of overlapping indels, there are *N *positions at which an indel occurred and *L*_*b *_- *N *positions that did not give rise to indels. Therefore,

f(Aln|B,t)=(e−λt)Lb−N(1−e−λt)N∏g=1Nf(kg)
 MathType@MTEF@5@5@+=feaafiart1ev1aaatCvAUfKttLearuWrP9MDH5MBPbIqV92AaeXatLxBI9gBaebbnrfifHhDYfgasaacH8akY=wiFfYdH8Gipec8Eeeu0xXdbba9frFj0=OqFfea0dXdd9vqai=hGuQ8kuc9pgc9s8qqaq=dirpe0xb9q8qiLsFr0=vr0=vr0dc8meaabaqaciaacaGaaeqabaqabeGadaaakeaacqWGMbGzcqGGOaakcqWGbbqqieGacqWFSbaBcqWFUbGBcqGG8baFcqWGcbGqcqGGSaalcqWG0baDcqGGPaqkcqGH9aqpdaqadaqaaiabdwgaLnaaCaaaleqabaGaeyOeI0ccciGae43UdWMaemiDaqhaaaGccaGLOaGaayzkaaWaaWbaaSqabeaacqWGmbatdaWgaaadbaGaemOyaigabeaaliabgkHiTiabd6eaobaakmaabmaabaGaeGymaeJaeyOeI0Iaemyzau2aaWbaaSqabeaacqGHsislcqGF7oaBcqWG0baDaaaakiaawIcacaGLPaaadaahaaWcbeqaaiabd6eaobaakmaarahabaGaemOzayMaeiikaGIaem4AaS2aaSbaaSqaaiabdEgaNbqabaGccqGGPaqkaSqaaiabdEgaNjabg2da9iabigdaXaqaaiabd6eaobqdcqGHpis1aaaa@5C73@

where *N *is the number of indels in the alignment, f(kg)=kg−z/ζ(z)
 MathType@MTEF@5@5@+=feaafiart1ev1aaatCvAUfKttLearuWrP9MDH5MBPbIqV92AaeXatLxBI9gBaebbnrfifHhDYfgasaacH8akY=wiFfYdH8Gipec8Eeeu0xXdbba9frFj0=OqFfea0dXdd9vqai=hGuQ8kuc9pgc9s8qqaq=dirpe0xb9q8qiLsFr0=vr0=vr0dc8meaabaqaciaacaGaaeqabaqabeGadaaakeaacqWGMbGzcqGGOaakcqWGRbWAdaWgaaWcbaGaem4zaCgabeaakiabcMcaPiabg2da9iabdUgaRnaaDaaaleaacqWGNbWzaeaacqGHsislcqWG6bGEaaGccqGGVaWliiGacqWF2oGEcqGGOaakcqWG6bGEcqGGPaqkaaa@3ED5@ is the probability that an indel has a length of size *k*_*g*_, and *λ *is the instantaneous rate of indel formation per unit branch length. Putting this all together,

f(Aln,A,B,t)=e−λtLb4La+Lb(1+3e−4t/3)M(1−e−4t/3)R×(e−λt)−N(1−e−λt)Ne−t/θθ∏g=1Nf(kg)
 MathType@MTEF@5@5@+=feaafiart1ev1aaatCvAUfKttLearuWrP9MDH5MBPbIqV92AaeXatLxBI9gBaebbnrfifHhDYfgasaacH8akY=wiFfYdH8Gipec8Eeeu0xXdbba9frFj0=OqFfea0dXdd9vqai=hGuQ8kuc9pgc9s8qqaq=dirpe0xb9q8qiLsFr0=vr0=vr0dc8meaabaqaciaacaGaaeqabaqabeGadaaakeaacqWGMbGzcqGGOaakcqWGbbqqieGacqWFSbaBcqWFUbGBcqGGSaalcqWGbbqqcqGGSaalcqWGcbGqcqGGSaalcqWG0baDcqGGPaqkcqGH9aqpdaWcaaqaaiabdwgaLnaaCaaaleqabaGaeyOeI0ccciGae43UdWMaemiDaqNaemitaW0aaSbaaWqaaiabdkgaIbqabaaaaaGcbaGaeGinaqZaaWbaaSqabeaacqWGmbatdaWgaaadbaGaemyyaegabeaaliabgUcaRiabdYeamnaaBaaameaacqWGIbGyaeqaaaaaaaGcdaqadaqaaiabigdaXiabgUcaRiabiodaZiabdwgaLnaaCaaaleqabaGaeyOeI0IaeGinaqJaemiDaqNaei4la8IaeG4mamdaaaGccaGLOaGaayzkaaWaaWbaaSqabeaacqWGnbqtaaGcdaqadaqaaiabigdaXiabgkHiTiabdwgaLnaaCaaaleqabaGaeyOeI0IaeGinaqJaemiDaqNaei4la8IaeG4mamdaaaGccaGLOaGaayzkaaWaaWbaaSqabeaacqWGsbGuaaGccqGHxdaTdaqadaqaaiabdwgaLnaaCaaaleqabaGaeyOeI0Iae43UdWMaemiDaqhaaaGccaGLOaGaayzkaaWaaWbaaSqabeaacqGHsislcqWGobGtaaGcdaqadaqaaiabigdaXiabgkHiTiabdwgaLnaaCaaaleqabaGaeyOeI0Iae43UdWMaemiDaqhaaaGccaGLOaGaayzkaaWaaWbaaSqabeaacqWGobGtaaGcdaWcaaqaaiabdwgaLnaaCaaaleqabaGaeyOeI0IaemiDaqNaei4la8Iae4hUdehaaaGcbaGae4hUdehaamaarahabaGaemOzayMaeiikaGIaem4AaS2aaSbaaSqaaiabdEgaNbqabaGccqGGPaqkaSqaaiabdEgaNjabg2da9iabigdaXaqaaiabd6eaobqdcqGHpis1aaaa@8CDF@

For simplicity we will not integrate Equation 7 to find *f*(*Aln*, *A*, *B*). Instead, we will approximate it based on the mean value of *t*:

*f *(*Aln*, *A*, *B*) ≈ *f *(*Aln*, *A*, *B*|*t *= t¯
 MathType@MTEF@5@5@+=feaafiart1ev1aaatCvAUfKttLearuWrP9MDH5MBPbIqV92AaeXatLxBI9gBaebbnrfifHhDYfgasaacH8akY=wiFfYdH8Gipec8Eeeu0xXdbba9frFj0=OqFfea0dXdd9vqai=hGuQ8kuc9pgc9s8qqaq=dirpe0xb9q8qiLsFr0=vr0=vr0dc8meaabaqaciaacaGaaeqabaqabeGadaaakeaacuWG0baDgaqeaaaa@2E35@) = *f *(*Aln*, *A*, *B*, *t *= t¯
 MathType@MTEF@5@5@+=feaafiart1ev1aaatCvAUfKttLearuWrP9MDH5MBPbIqV92AaeXatLxBI9gBaebbnrfifHhDYfgasaacH8akY=wiFfYdH8Gipec8Eeeu0xXdbba9frFj0=OqFfea0dXdd9vqai=hGuQ8kuc9pgc9s8qqaq=dirpe0xb9q8qiLsFr0=vr0=vr0dc8meaabaqaciaacaGaaeqabaqabeGadaaakeaacuWG0baDgaqeaaaa@2E35@)/*f *(*t *= t¯
 MathType@MTEF@5@5@+=feaafiart1ev1aaatCvAUfKttLearuWrP9MDH5MBPbIqV92AaeXatLxBI9gBaebbnrfifHhDYfgasaacH8akY=wiFfYdH8Gipec8Eeeu0xXdbba9frFj0=OqFfea0dXdd9vqai=hGuQ8kuc9pgc9s8qqaq=dirpe0xb9q8qiLsFr0=vr0=vr0dc8meaabaqaciaacaGaaeqabaqabeGadaaakeaacuWG0baDgaqeaaaa@2E35@)

Upon removing factors that are constant for sequences *A *and *B *we get the likelihood for the alignment *Aln *given sequence pair *A *and *B *and parameters *λ*, *θ*, and *z*:

L(Aln|A,B)=(1+3e−4θ/3)M(1−e−4θ/3)R×∏g=1Neλθ−1ζ(z)kg−z     (9)
 MathType@MTEF@5@5@+=feaafiart1ev1aaatCvAUfKttLearuWrP9MDH5MBPbIqV92AaeXatLxBI9gBaebbnrfifHhDYfgasaacH8akY=wiFfYdH8Gipec8Eeeu0xXdbba9frFj0=OqFfea0dXdd9vqai=hGuQ8kuc9pgc9s8qqaq=dirpe0xb9q8qiLsFr0=vr0=vr0dc8meaabaqaciaacaGaaeqabaqabeGadaaakeaacqWGmbatcqGGOaakcqWGbbqqieGacqWFSbaBcqWFUbGBcqGG8baFcqWGbbqqcqGGSaalcqWGcbGqcqGGPaqkcqGH9aqpdaqadaqaaiabigdaXiabgUcaRiabiodaZiabdwgaLnaaCaaaleqabaGaeyOeI0IaeGinaqdcciGae4hUdeNaei4la8IaeG4mamdaaaGccaGLOaGaayzkaaWaaWbaaSqabeaacqWGnbqtaaGcdaqadaqaaiabigdaXiabgkHiTiabdwgaLnaaCaaaleqabaGaeyOeI0IaeGinaqJae4hUdeNaei4la8IaeG4mamdaaaGccaGLOaGaayzkaaWaaWbaaSqabeaacqWGsbGuaaGccqGHxdaTdaqeWbqaamaalaaabaGaemyzau2aaWbaaSqabeaacqGF7oaBcqGF4oqCaaGccqGHsislcqaIXaqmaeaacqGF2oGEcqGGOaakcqWG6bGEcqGGPaqkaaaaleaacqWGNbWzcqGH9aqpcqaIXaqmaeaacqWGobGta0Gaey4dIunakiabdUgaRnaaDaaaleaacqWGNbWzaeaacqGHsislcqWG6bGEaaGccaWLjaGaaCzcamaabmaabaGaeGyoaKdacaGLOaGaayzkaaaaaa@6EE1@

The alignment is quantified by the number of matches (*M*), the number of mismatches (*R*), and the set of gap lengths, (*k*_1 _. . . *k*_*N*_). The likelihood of an alignment depends on three evolutionary parameters: the rate of indel formation per unit branch length (*λ*), the average branch length between two sequences (*θ*), and the "slope" of the power law (*z*). Furthermore, the log-likelihood is

ln⁡L(Aln|A,B)=Mln⁡(1+3e−4θ/3)+Rln⁡(1−e−4θ/3)+∑g=1N[ln⁡(eλθ−1)−ln⁡ζ(z)−zln⁡kg]     (10)
 MathType@MTEF@5@5@+=feaafiart1ev1aaatCvAUfKttLearuWrP9MDH5MBPbIqV92AaeXatLxBI9gBaebbnrfifHhDYfgasaacH8akY=wiFfYdH8Gipec8Eeeu0xXdbba9frFj0=OqFfea0dXdd9vqai=hGuQ8kuc9pgc9s8qqaq=dirpe0xb9q8qiLsFr0=vr0=vr0dc8meaabaqaciaacaGaaeqabaqabeGadaaakeaacyGGSbaBcqGGUbGBcqWGmbatcqGGOaakcqWGbbqqieGacqWFSbaBcqWFUbGBcqGG8baFcqWGbbqqcqGGSaalcqWGcbGqcqGGPaqkcqGH9aqpcqWGnbqtcyGGSbaBcqGGUbGBdaqadaqaaiabigdaXiabgUcaRiabiodaZiabdwgaLnaaCaaaleqabaGaeyOeI0IaeGinaqdcciGae4hUdeNaei4la8IaeG4mamdaaaGccaGLOaGaayzkaaGaey4kaSIaemOuaiLagiiBaWMaeiOBa42aaeWaaeaacqaIXaqmcqGHsislcqWGLbqzdaahaaWcbeqaaiabgkHiTiabisda0iab+H7aXjabc+caViabiodaZaaaaOGaayjkaiaawMcaaiabgUcaRmaaqahabaWaamWaaeaacyGGSbaBcqGGUbGBcqGGOaakcqWGLbqzdaahaaWcbeqaaiab+T7aSjab+H7aXbaakiabgkHiTiabigdaXiabcMcaPiabgkHiTiGbcYgaSjabc6gaUjab+z7a6jabcIcaOiabdQha6jabcMcaPiabgkHiTiabdQha6jGbcYgaSjabc6gaUjabdUgaRnaaBaaaleaacqWGNbWzaeqaaaGccaGLBbGaayzxaaaaleaacqWGNbWzcqGH9aqpcqaIXaqmaeaacqWGobGta0GaeyyeIuoakiaaxMaacaWLjaWaaeWaaeaacqaIXaqmcqaIWaamaiaawIcacaGLPaaaaaa@8433@

### B. Gap costs

As developed by Smith et al. [13] and extended by Holmes and Durban [16] and below, a maximum likelihood search can be converted to a minimum cost search with shifting and scaling. Based on a statistical model, the scores of "matches" of type *i*, *α*_*i*_, and the penalties of gaps of length *k*, *w*_*k*_, can be used to calculate the alignment with maximum log-likelihood:

l=max⁡{∑αiηi−∑wkΔk}     (11)
 MathType@MTEF@5@5@+=feaafiart1ev1aaatCvAUfKttLearuWrP9MDH5MBPbIqV92AaeXatLxBI9gBaebbnrfifHhDYfgasaacH8akY=wiFfYdH8Gipec8Eeeu0xXdbba9frFj0=OqFfea0dXdd9vqai=hGuQ8kuc9pgc9s8qqaq=dirpe0xb9q8qiLsFr0=vr0=vr0dc8meaabaqaciaacaGaaeqabaqabeGadaaakeaacqWGSbaBcqGH9aqpcyGGTbqBcqGGHbqycqGG4baEdaGadeqaamaaqaeabaacciGae8xSde2aaSbaaSqaaiabdMgaPbqabaGccqWF3oaAdaWgaaWcbaGaemyAaKgabeaakiabgkHiTmaaqaeabaGaem4DaC3aaSbaaSqaaiabdUgaRbqabaGccqqHuoardaWgaaWcbaGaem4AaSgabeaaaeqabeqdcqGHris5aaWcbeqab0GaeyyeIuoaaOGaay5Eaiaaw2haaiaaxMaacaWLjaWaaeWaaeaacqaIXaqmcqaIXaqmaiaawIcacaGLPaaaaaa@4B98@

where *η*_*i *_is the number of residue matches of type *i *and Δ_*k *_is the number of gaps of length *k*. A minimum cost analog of Equation 11 is

d=min⁡{∑βiηi+∑G(k)Δk}     (11)
 MathType@MTEF@5@5@+=feaafiart1ev1aaatCvAUfKttLearuWrP9MDH5MBPbIqV92AaeXatLxBI9gBaebbnrfifHhDYfgasaacH8akY=wiFfYdH8Gipec8Eeeu0xXdbba9frFj0=OqFfea0dXdd9vqai=hGuQ8kuc9pgc9s8qqaq=dirpe0xb9q8qiLsFr0=vr0=vr0dc8meaabaqaciaacaGaaeqabaqabeGadaaakeaacqWGKbazcqGH9aqpcyGGTbqBcqGGPbqAcqGGUbGBdaGadeqaamaaqaeabaacciGae8NSdi2aaSbaaSqaaiabdMgaPbqabaGccqWF3oaAdaWgaaWcbaGaemyAaKgabeaakiabgUcaRmaaqaeabaGaem4raCKaeiikaGIaem4AaSMaeiykaKIaeuiLdq0aaSbaaSqaaiabdUgaRbqabaaabeqab0GaeyyeIuoaaSqabeqaniabggHiLdaakiaawUhacaGL9baacaWLjaGaaCzcamaabmaabaGaeGymaeJaeGymaedacaGLOaGaayzkaaaaaa@4C97@

To begin constructing the minimum cost analog, let *β*_*i *_= (*x *- *α*_*i*_)/*y *be the cost of a match of type *i*, therefore

−l=min⁡{−∑αiηi+∑wkΔk}=min⁡{∑(yβi−x)ηi+∑wkΔk}=ymin⁡{∑βiηi−xy∑ηi+∑wkyΔk}     (12)
 MathType@MTEF@5@5@+=feaafiart1ev1aaatCvAUfKttLearuWrP9MDH5MBPbIqV92AaeXatLxBI9gBaebbnrfifHhDYfgasaacH8akY=wiFfYdH8Gipec8Eeeu0xXdbba9frFj0=OqFfea0dXdd9vqai=hGuQ8kuc9pgc9s8qqaq=dirpe0xb9q8qiLsFr0=vr0=vr0dc8meaabaqaciaacaGaaeqabaqabeGadaaakeaacqGHsislcqWGSbaBcqGH9aqpcyGGTbqBcqGGPbqAcqGGUbGBdaGadeqaaiabgkHiTmaaqaeabaacciGae8xSde2aaSbaaSqaaiabdMgaPbqabaGccqWF3oaAdaWgaaWcbaGaemyAaKgabeaakiabgUcaRmaaqaeabaGaem4DaC3aaSbaaSqaaiabdUgaRbqabaGccqqHuoardaWgaaWcbaGaem4AaSgabeaaaeqabeqdcqGHris5aaWcbeqab0GaeyyeIuoaaOGaay5Eaiaaw2haaiabg2da9iGbc2gaTjabcMgaPjabc6gaUnaacmqabaWaaabqaeaacqGGOaakcqWG5bqEcqWFYoGydaWgaaWcbaGaemyAaKgabeaakiabgkHiTiabdIha4jabcMcaPiab=D7aOnaaBaaaleaacqWGPbqAaeqaaOGaey4kaSYaaabqaeaacqWG3bWDdaWgaaWcbaGaem4AaSgabeaakiabfs5aenaaBaaaleaacqWGRbWAaeqaaaqabeqaniabggHiLdaaleqabeqdcqGHris5aaGccaGL7bGaayzFaaGaeyypa0JaemyEaKNagiyBa0MaeiyAaKMaeiOBa42aaiWabeaadaaeabqaaiab=j7aInaaBaaaleaacqWGPbqAaeqaaOGae83TdG2aaSbaaSqaaiabdMgaPbqabaGccqGHsisldaWcaaqaaiabdIha4bqaaiabdMha5baadaaeabqaaiab=D7aOnaaBaaaleaacqWGPbqAaeqaaOGaey4kaSYaaabqaeaadaWcaaqaaiabdEha3naaBaaaleaacqWGRbWAaeqaaaGcbaGaemyEaKhaaiabfs5aenaaBaaaleaacqWGRbWAaeqaaaqabeqaniabggHiLdaaleqabeqdcqGHris5aaWcbeqab0GaeyyeIuoaaOGaay5Eaiaaw2haaiaaxMaacaWLjaWaaeWaaeaacqaIXaqmcqaIYaGmaiaawIcacaGLPaaaaaa@90C5@

The lengths of the sequences being aligned, *n *and *m*, can be related to the alignment itself via the equation *n *+ *m *= 2∑*η*_*i *_+ ∑*k*Δ_*k*_. Using this relationship, Equation 12 can be expressed as

−l=ymin⁡{∑βiηi−x2y(n+m−∑kΔk)+∑wkyΔk}=ymin⁡{∑βiηi+x2y∑kΔk+∑wkyΔk}−x(n+m)2=ymin⁡{∑βiηi+∑(xk2y+wky)Δk}−x(n+m)2=ymin⁡{∑βiηi+∑G(k)Δk}−x(n+m)2
 MathType@MTEF@5@5@+=feaafiart1ev1aaatCvAUfKttLearuWrP9MDH5MBPbIqV92AaeXatLxBI9gBaebbnrfifHhDYfgasaacH8akY=wiFfYdH8Gipec8Eeeu0xXdbba9frFj0=OqFfea0dXdd9vqai=hGuQ8kuc9pgc9s8qqaq=dirpe0xb9q8qiLsFr0=vr0=vr0dc8meaabaqaciaacaGaaeqabaqabeGadaaakeaafaqadeabbaaaaeaacqGHsislcqWGSbaBcqGH9aqpcqWG5bqEcyGGTbqBcqGGPbqAcqGGUbGBdaGadeqaamaaqaeabaacciGae8NSdi2aaSbaaSqaaiabdMgaPbqabaGccqWF3oaAdaWgaaWcbaGaemyAaKgabeaakiabgkHiTmaalaaabaGaemiEaGhabaGaeGOmaiJaemyEaKhaamaabmaabaGaemOBa4Maey4kaSIaemyBa0MaeyOeI0YaaabqaeaacqWGRbWAcqqHuoardaWgaaWcbaGaem4AaSgabeaaaeqabeqdcqGHris5aaGccaGLOaGaayzkaaGaey4kaSYaaabqaeaadaWcaaqaaiabdEha3naaBaaaleaacqWGRbWAaeqaaaGcbaGaemyEaKhaaiabfs5aenaaBaaaleaacqWGRbWAaeqaaaqabeqaniabggHiLdaaleqabeqdcqGHris5aaGccaGL7bGaayzFaaaabaGaeyypa0JaemyEaKNagiyBa0MaeiyAaKMaeiOBa42aaiWabeaadaaeabqaaiab=j7aInaaBaaaleaacqWGPbqAaeqaaOGae83TdG2aaSbaaSqaaiabdMgaPbqabaGccqGHRaWkdaWcaaqaaiabdIha4bqaaiabikdaYiabdMha5baadaaeabqaaiabdUgaRjabfs5aenaaBaaaleaacqWGRbWAaeqaaaqabeqaniabggHiLdGccqGHRaWkdaaeabqaamaalaaabaGaem4DaC3aaSbaaSqaaiabdUgaRbqabaaakeaacqWG5bqEaaGaeuiLdq0aaSbaaSqaaiabdUgaRbqabaaabeqab0GaeyyeIuoaaSqabeqaniabggHiLdaakiaawUhacaGL9baacqGHsisldaWcaaqaaiabdIha4jabcIcaOiabd6gaUjabgUcaRiabd2gaTjabcMcaPaqaaiabikdaYaaaaeaacqGH9aqpcqWG5bqEcyGGTbqBcqGGPbqAcqGGUbGBdaGadeqaamaaqaeabaGae8NSdi2aaSbaaSqaaiabdMgaPbqabaGccqWF3oaAdaWgaaWcbaGaemyAaKgabeaakiabgUcaRmaaqaeabaWaaeWaaeaadaWcaaqaaiabdIha4jabdUgaRbqaaiabikdaYiabdMha5baacqGHRaWkdaWcaaqaaiabdEha3naaBaaaleaacqWGRbWAaeqaaaGcbaGaemyEaKhaaaGaayjkaiaawMcaaaWcbeqab0GaeyyeIuoakiabfs5aenaaBaaaleaacqWGRbWAaeqaaaqabeqaniabggHiLdaakiaawUhacaGL9baacqGHsisldaWcaaqaaiabdIha4jabcIcaOiabd6gaUjabgUcaRiabd2gaTjabcMcaPaqaaiabikdaYaaaaeaacqGH9aqpcqWG5bqEcyGGTbqBcqGGPbqAcqGGUbGBdaGadeqaamaaqaeabaGae8NSdi2aaSbaaSqaaiabdMgaPbqabaGccqWF3oaAdaWgaaWcbaGaemyAaKgabeaakiabgUcaRmaaqaeabaGaem4raCKaeiikaGIaem4AaSMaeiykaKIaeuiLdq0aaSbaaSqaaiabdUgaRbqabaaabeqab0GaeyyeIuoaaSqabeqaniabggHiLdaakiaawUhacaGL9baacqGHsisldaWcaaqaaiabdIha4jabcIcaOiabd6gaUjabgUcaRiabd2gaTjabcMcaPaqaaiabikdaYaaaaaaaaa@DC8D@

From this it can be clearly seen that *d *= min{∑*β*_*i*_*η*_*i *_+ ∑*G *(*k*)Δ_*k*_} maximizes the likelihood of the alignment, where *G *(*k*) = (*xk*/2 + *w*_*k*_)/*y *is the cost of a gap of length *k*. Applying this method to Equation 10 such that the cost of a match is 0 and the cost of a mismatch is 1 produces the following equation for a gap cost:

G(k)=ln⁡ζ(z)−ln⁡(eλθ−1)+ln⁡(1+3e−4θ/3)k/2+zln⁡kln⁡(1+3e−4θ/3)−ln⁡(1−e−4θ/3)     (13)
 MathType@MTEF@5@5@+=feaafiart1ev1aaatCvAUfKttLearuWrP9MDH5MBPbIqV92AaeXatLxBI9gBaebbnrfifHhDYfgasaacH8akY=wiFfYdH8Gipec8Eeeu0xXdbba9frFj0=OqFfea0dXdd9vqai=hGuQ8kuc9pgc9s8qqaq=dirpe0xb9q8qiLsFr0=vr0=vr0dc8meaabaqaciaacaGaaeqabaqabeGadaaakeaacqWGhbWrcqGGOaakcqWGRbWAcqGGPaqkcqGH9aqpdaWcaaqaaiGbcYgaSjabc6gaUHGaciab=z7a6jabcIcaOiabdQha6jabcMcaPiabgkHiTiGbcYgaSjabc6gaUjabcIcaOiabdwgaLnaaCaaaleqabaGae83UdWMae8hUdehaaOGaeyOeI0IaeGymaeJaeiykaKIaey4kaSIagiiBaWMaeiOBa4MaeiikaGIaeGymaeJaey4kaSIaeG4mamJaemyzau2aaWbaaSqabeaacqGHsislcqaI0aancqWF4oqCcqGGVaWlcqaIZaWmaaGccqGGPaqkcqWGRbWAcqGGVaWlcqaIYaGmcqGHRaWkcqWG6bGEcyGGSbaBcqGGUbGBcqWGRbWAaeaacyGGSbaBcqGGUbGBcqGGOaakcqaIXaqmcqGHRaWkcqaIZaWmcqWGLbqzdaahaaWcbeqaaiabgkHiTiabisda0iab=H7aXjabc+caViabiodaZaaakiabcMcaPiabgkHiTiGbcYgaSjabc6gaUjabcIcaOiabigdaXiabgkHiTiabdwgaLnaaCaaaleqabaGaeyOeI0IaeGinaqJae8hUdeNaei4la8IaeG4mamdaaOGaeiykaKcaaiaaxMaacaWLjaWaaeWaaeaacqaIXaqmcqaIZaWmaiaawIcacaGLPaaaaaa@7FC3@
